# Knowledge and Prevalence of Bruxism and Its Association With Stress Among Clinical‐Year Dental and Medical Students at the University of Ghana

**DOI:** 10.1155/ijod/2469933

**Published:** 2026-10-15

**Authors:** Shadrach Terifu-nenom Daabaar, Joseph Kwasi Nimako-Boateng, Daniel Tormeti, Alfred Dai-Kosi, Josephine Sackeyfio, Edmond Kubi Appiah, Edward Ohene-Marfo

**Affiliations:** ^1^ Department of Community and Preventive Dentistry University of Ghana Dental School College of Health Sciences University of Ghana Accra Ghana ug.edu.gh; ^2^ Department of GI Nutrition and Hepatology Children’s Hospital of Philadelphia Philadelphia USA chop.edu; ^3^ University of Arkansas for Medical Sciences Little Rock Arkansas USA uams.edu

**Keywords:** bruxism, Ghana, health knowledge attitudes practice, prevalence, sleep bruxism, stress psychological, students dental, students medical

## Abstract

**Background:**

Bruxism is a repetitive jaw‐muscle activity characterised by clenching or grinding of the teeth and/or by bracing or thrusting of the mandible. Current international consensus regards it as a motor behaviour rather than a disorder, and one that may act as a risk factor for tooth wear and temporomandibular disorders; psychosocial stress is among its most consistently reported associated factors. Clinical‐year dental and medical students face intense academic and clinical pressure that may predispose them to both stress and bruxism, yet no published Ghanaian data on this relationship previously existed.

**Methods:**

A cross‐sectional study was conducted among clinical‐year (4th–6th year) dental and medical students at the University of Ghana, Korle Bu campus. A stratified sample of 291 students, proportionally allocated by course and clinical year, completed a self‐administered online questionnaire comprising a 12‐point bruxism knowledge module, a 10‐item bruxism symptom screening module and the 30‐item Perceived Stress Questionnaire (PSQ). Prevalence estimates are reported with 95% confidence intervals (CIs). Associations were examined using chi‐square tests, multivariable linear regression, and Pearson and Spearman correlation coefficients, at *α* = 0.05.

**Results:**

Participants had a mean age of 23.6 ± 2.3 years; 55.0% were male, and 85.2% were medical students. Only 19.6% (95% CI: 15.4%–24.5%) demonstrated good knowledge of bruxism, with dental students (48.8% vs. 14.5%, *p* < 0.001) and final‐year students (*p* = 0.04) significantly more knowledgeable. Self‐reported awareness of awake bruxism was 36.1% (95% CI: 30.8%–41.7%) and of sleep bruxism 14.4% (95% CI: 10.9%–18.9%); on the composite 10‐item screening score, 9.3% (95% CI: 6.5%–13.2%) of students met the threshold for subject‐based (self‐reported) bruxism, with no significant socio‐demographic differences. High perceived stress was recorded in 8.9% (95% CI: 6.2%–12.8%) of students. Stress correlated significantly, though weakly, with bruxism symptom scores (Pearson’s *r* = 0.25, 95% CI: 0.14–0.35, *p* < 0.001; Spearman’s *ρ* = 0.212, *p* = 0.0003 (*p* < 0.001).

**Conclusion:**

Knowledge of bruxism is markedly poorer among medical than dental students, and among junior compared with final‐year trainees. Self‐reported bruxism symptoms are common despite a low rate of high perceived stress, and stress shows a modest but significant positive association with bruxism. Curricular bruxism education for medical students and proactive stress‐reduction initiatives are warranted.

## 1. Introduction

Bruxism is a repetitive jaw‐muscle activity characterised by clenching or grinding of the teeth and/or by bracing or thrusting of the mandible, with two distinct circadian manifestations: sleep bruxism, a masticatory muscle activity during sleep that is rhythmic (phasic) or non‐rhythmic (tonic), and awake bruxism, a masticatory muscle activity during wakefulness characterised by repetitive or sustained tooth contact and/or by bracing or thrusting of the mandible [1, 2]. The 2025 update of the international consensus reaffirms that bruxism is a motor behaviour rather than a disorder, removes the earlier qualifier ‘in otherwise healthy individuals’, and frames bruxism as a factor that, depending on clinical context, may be a risk factor for adverse oral outcomes such as tooth wear, dentine hypersensitivity and masticatory muscle or temporomandibular joint pain, a protective factor (for example in sleep‐related breathing disorders), or a neutral factor [2, 3]. The same update replaces the earlier hierarchical grading of ‘possible’, ‘probable’ and ‘definite’ bruxism with three complementary modes of assessment: subject‐based (self‐report), clinically based (examination) and device‐based (electromyography or polysomnography) [2].

Although early theories attributed bruxism to malocclusion and occlusal interference, this view has largely been abandoned in favour of a multifactorial model encompassing psychosocial stress, anxiety, central and autonomic nervous system mechanisms, certain medications and lifestyle factors such as caffeine, alcohol and tobacco use [4, 5]. Reported bruxism prevalence varies widely by population and diagnostic method, with systematic reviews placing the global adult range at roughly 8%–31% [6] and university and health‐professional student populations consistently reporting prevalence at or above the upper end of this range—reflecting heightened academic and clinical pressure [7, 8].

Clinical‐year dental and medical training is a period of sustained academic, clinical and evaluative demand, and the orofacial region is a recognised site for the physical expression of psychological stress, including bruxism, aphthous ulceration and burning mouth symptoms [9]. Globally, studies among university and health professional students have reported self‐reported bruxism prevalence of ~31%–42% [8, 10–12]. Several of these studies have also demonstrated a significant association between perceived stress or psychological distress and bruxism: Cavallo et al. [8] found a positive correlation between Perceived Stress Scale scores and awake bruxism among Italian university students; Kolak et al. [12] reported higher stress, anxiety and depression scores and elevated salivary cortisol among Serbian dental students with probable bruxism; and Osses‐Anguita et al. [13] linked shifts in awake and sleep bruxism prevalence among first‐year university students to changes in anxiety and coping style across the COVID‐19 pandemic. The strength of the association nonetheless varies with the stress instrument and bruxism assessment method used, and it has been demonstrated more consistently for awake than for sleep bruxism.

Within West Africa, published epidemiological data on bruxism remain scarce; the only comparable estimate identified was a 24.7% self‐reported prevalence among undergraduates at a Nigerian university [14]. At the time of this study, no peer‐reviewed Ghanaian data on bruxism prevalence, knowledge or its relationship with stress were available. This study therefore aimed to assess the knowledge and prevalence of bruxism, the level of perceived stress, and the association between the two among clinical‐year dental and medical students at the University of Ghana.

## 2. Methods

### 2.1. Study Design and Setting

This was a cross‐sectional, descriptive and observational study conducted at the Korle Bu campus of the University of Ghana, which houses the College of Health Sciences, including the Medical and Dental Schools and their associated clinical‐year departments.

### 2.2. Study Population, Sampling and Recruitment

The study population comprised clinical‐year (4th, 5th and final/6th year) dental and medical students. Eligible participants were clinical‐year students who provided consent; those who declined were excluded. The minimum sample size was calculated using Cochran’s formula at a 95% confidence level and 5% margin of error using an expected prevalence of 24.7% drawn from the Nigerian undergraduate study by Azodo and Ojehanon [14], yielding a minimum requirement of 291. A stratified sampling technique, with strata defined by course of study and clinical year, was used to allocate proportional quotas; within each stratum, a random‐number procedure in Microsoft Excel was used to select participants from the class lists, who were then invited via WhatsApp links to an online questionnaire.

### 2.3. Data Collection Instrument

Data were collected through a self‐administered structured questionnaire delivered via Google Forms. The instrument was developed by the investigators for this study, reviewed for content validity by specialists, and piloted among clinical‐year students at the Kumasi School of Medicine and Dentistry. The full instrument is provided in File S1. It comprised four sections:

Section A—socio‐demographic characteristics: age, sex, course of study (dentistry or medicine) and clinical year.
Section B—knowledge of bruxism (12 points). Participants were first asked whether they had ever heard the term ‘bruxism’ and, if so, from which sources (school, mass media, family/friends, health professional or other). Knowledge was then assessed with multiple‐response items on (i) the definition of bruxism, (ii) its causes, (iii) its consequences and (iv) its treatment options. One point was awarded for each correct response and no points for incorrect or distractor options, giving a maximum score of 12. Participants who had never heard the term were assigned a score of zero. A score of ≥6 (≥50%) was classified as ‘good knowledge’ and <6 as ‘poor knowledge’.
Section C—bruxism symptoms and signs (10 items). Ten yes/no items captured self‐reported awareness of awake tooth grinding or clenching; awareness of, or having been told about, tooth grinding during sleep; jaw fatigue or soreness on waking; clicking of the temporomandibular joint and a ridge (linea alba) on the buccal mucosa. Each affirmative response scored one point (range 0–10). Consistent with the 2025 consensus framework, this module constitutes a subject‐based (self‐report) assessment of bruxism [2]; a score of ≥4 was taken as the threshold for a positive subject‐based screen (equivalent to ‘possible bruxism’ under the 2018 grading system).
Section D—perceived stress. The 30‐item Perceived Stress Questionnaire (PSQ), a validated instrument developed by Levenstein et al. [15] to quantify the subjective experience of stressful life events and circumstances, was administered in its general (past 2 years) form. Each item is rated on a 4‐point scale (1 = almost never, 2 = sometimes, 3 = often and 4 = usually); eight positively worded items are reverse‐scored. The PSQ index was computed as (raw score − 30)/90, yielding a value between 0 and 1, with higher values indicating greater perceived stress. High perceived stress was defined as a PSQ index.

### 2.4. Statistical Analysis

Data were exported from Google Forms to Microsoft Excel and analysed in IBM SPSS Statistics version 22. Continuous variables were summarised as means and standard deviations, and categorical variables as frequencies and percentages with 95% confidence intervals (CIs) computed by the Wilson score method. Chi‐square tests (or Fisher’s exact test where expected cell counts were <5) assessed associations between socio‐demographic characteristics and knowledge, bruxism, or stress outcomes; multivariable linear regression examined predictors of the knowledge score; and the relationship between the PSQ index and the bruxism symptom score was examined by correlation. The distributions of both scores were assessed for normality using the Shapiro–Wilk test, with inspection of histograms and Q–Q plots. Because the bruxism symptom score was right‐skewed with a high proportion of zero scores/was not normally distributed, Spearman’s rank correlation coefficient (*ρ*) was used as the primary measure of association, with Pearson’s *r* reported alongside for comparability with prior studies; the 95% CI for *r* was obtained by Fisher’s *z*‐transformation. Significance was set at *p* < 0.05.

### 2.5. Ethical Considerations

Ethical clearance was obtained from the Ethical Review Board of the College of Health Sciences, University of Ghana. Participants were informed of study objectives, and electronic informed consent was obtained prior to participation; responses were anonymous, and confidentiality was assured throughout.

## 3. Results

### 3.1. Participant Characteristics

Two hundred and ninety‐one clinical‐year students participated (mean age 23.6 ± 2.3 years; 70.1% aged 21–24 years). Males comprised 55.0% of the sample, and medical students comprised 85.2%; all three clinical years were broadly represented (36.1% first clinical year, 33.3% second and 30.6% final year) (Table 1).

**Table 1:** Socio‐demographic characteristics of participants (*N* = 291).

Characteristic	*n*	%	95% CI
Mean age (years)	23.6 ± 2.3	—	—
Male sex	160	55.0	49.2–60.6
Medical students	248	85.2	80.7–88.8
Dental students	43	14.8	11.2–19.3
1st clinical year	105	36.1	30.8–41.7
2nd clinical year	97	33.3	28.2–38.9
Final clinical year	89	30.6	25.6–36.1

*Note:* CI, confidence interval (Wilson score method).

### 3.2. Knowledge of Bruxism

Overall knowledge of bruxism was poor: 55.3% (95% CI: 49.6%–60.9%) of students had never heard the term, and only 19.6% (*n* = 57; 95% CI: 15.4%–24.5%) achieved a ‘good knowledge’ score. Among those familiar with the term, 32.6% could correctly define bruxism as encompassing both clenching and grinding, while a quarter (25.4%) and roughly a third (30.9%) reported knowing its causes and consequences, respectively; fewer (18.9%) reported knowledge of treatment options. School (56.3%) and mass media (54.7%) were the most‐cited information sources. Stress (90.7%), psychiatric problems (61.3%) and malocclusion (49.3%) were the most commonly perceived causes; tooth wear (94.4%) and myofascial pain (66.7%) were the most cited consequences (with arthritis of the jaw—51.1%—incorrectly believed to be a consequence); and dental guards/occlusal splints (87.0%) were the most recognised treatment. Good knowledge was significantly more prevalent among dental than medical students (48.8%, 95% CI: 34.6%–63.2% vs. 14.5%, 95% CI: 10.7%–19.4%; *p* < 0.001) and increased across clinical years (*p* = 0.04), but did not differ by sex (Table 2).

**Table 2:** Knowledge of bruxism by socio‐demographic characteristics (*N* = 291).

Variable	Category	Good knowledge, *n*/*N*	%	95% CI	*p*‐Value
Course	Dentistry	21/43	48.8	34.6–63.2	<0.001
	Medicine	36/248	14.5	10.7–19.4	—
Clinical year	First	15/105	14.3	8.9–22.2	0.04
	Second	17/97	17.5	11.2–26.3	—
	Final	25/89	19.1	19.8–38.2	—
Sex	Female	22/131	16.8	11.4–24.1	0.28
	Male	35/160	21.9	16.2–28.9	—

*Note:* Good knowledge = score ≥ 6/12. CI, confidence interval (Wilson score method). *p*‐Values from chi‐square tests.

In multivariable linear regression, dentistry as course of study remained the only significant independent predictor of knowledge score (*β* = 0.335, *p*  < 0.001); age, sex and clinical year were not independently significant.

### 3.3. Awareness and Self‐Reported Experience of Bruxism

Awareness of awake grinding or clenching was reported by 36.1% (95% CI: 30.8%–41.7%) of students, while 14.4% (95% CI: 10.9%–18.9%) were aware of, or had been told about, grinding during sleep. On the 10‐item composite symptom score, 27 students (9.3%, 95% CI: 6.5%–13.2%) reached the ≥ 4 threshold for a positive subject‐based bruxism screen. This proportion did not differ significantly by age, sex, course of study or clinical year (all *p* > 0.05) (Table 3).

**Table 3:** Self‐reported bruxism awareness, symptoms and subject‐based screening prevalence (*N* = 291).

Indicator	*n*	%	95% CI
Aware of awake grinding/clenching	105	36.1	30.8–41.7
Aware of sleep grinding	42	14.4	10.9–18.9
Linea alba on buccal mucosa	57	19.6	15.4–24.5
TMJ clicking	36	12.4	9.1–16.7
Jaw fatigue/soreness on waking	29	10.0	7.0–13.9
Composite screening score ≥ 4 (positive subject‐based screen)	27	9.3	6.5–13.2

*Note:* Terminology follows Verhoeff et al. [2]: ‘awake’ and ‘sleep’ bruxism denote state of consciousness. CI, confidence interval (Wilson score method). The remaining 5 items of the 10‐item score are listed in File S1.

### 3.4. Perceived Stress

Using the PSQ, 8.9% of students (*n* = 26; 95% CI: 6.2%–12.8%) recorded high perceived stress, with no significant association by age, sex, course of study or clinical year. The highest‐scoring stress items were feeling burdened by too many tasks (22.0% reporting this ‘usually’) and feeling under pressure from deadlines (13.4% ‘usually’).

### 3.5. Association Between Stress and Bruxism

The bruxism symptom score departed significantly from normality (Shapiro–Wilk *W* = 0.688, *p* < 0.001; median 1, IQR [0–2]), as did the PSQ index (*W* = 0.996, *p* = 0.644. Spearman’s rank correlation confirmed a statistically significant positive association between perceived stress and bruxism symptom score (*ρ* = 0.21, 95% CI: 0.10–0.32, *p* < 0.001), consistent in direction and magnitude with the Pearson correlation (*r* = 0.25, 95% CI: 0.14–0.35, *p* < 0.001) (Table 4): students with higher stress scores were more likely to report symptoms suggestive of bruxism.

**Table 4:** Correlation between perceived stress (PSQ index) and bruxism symptom score (*N* = 291).

Statistic	Coefficient	95% CI	*p*‐Value
Shapiro–Wilk W—bruxism symptom score	0.688	—	<0.001
Shapiro–Wilk W—PSQ index	0.996	—	0.644
Spearman’s *ρ*	0.21	0.10–0.32	<0.001
Pearson’s *r*	0.25	0.14–0.35	<0.001

*Note:* CI for Pearson’s *r* by Fisher’s *z*‐transformation; CI for Spearman’s *ρ* by (bootstrap [1000 resamples]/Fisher’s *z*).

## 4. Discussion

This study provides the first published assessment, to our knowledge, of bruxism knowledge, prevalence and its association with stress among Ghanaian clinical‐year dental and medical students. Three findings merit emphasis: a marked knowledge deficit, particularly among medical students; a sizeable gap between self‐reported awareness of bruxism behaviours and the proportion meeting a stricter composite screening threshold; and a statistically significant, if modest, link between stress and bruxism symptoms.

Consistent with prior comparative literature, dental students demonstrated substantially better knowledge of bruxism than their medical counterparts (48.8% vs. 14.5% good knowledge, *p* < 0.001), a pattern plausibly explained by bruxism’s explicit place in dental, but not medical, curricula. Even among dental students, however, surveys elsewhere have found that understanding of the multifactorial aetiology and of the range of management options remains incomplete [16, 17], and the wide CI around our dental student estimate (34.6%–63.2%) reflects the small size of that subgroup. Final‐year students also outperformed junior peers, consistent with cumulative clinical exposure. Notably, fewer than half of students who had heard of bruxism could define it correctly as encompassing both clenching and grinding—a stricter standard than some comparator studies have applied, which may explain why this study’s overall ‘good knowledge’ proportion (19.6%) sits below figures reported among general population samples elsewhere, where definitional criteria were broader [18].

The knowledge deficit among medical students has clinical implications that extend beyond the curriculum. In Ghana, as in much of sub‐Saharan Africa, the ratio of dentists to population is low and most people’s first, and often only, contact with the health system is through a physician or other non‐dental clinician. Medical practitioners are therefore well placed to recognise the early self‐reported and clinical indicators of bruxism—morning jaw fatigue, temporal headache, tooth wear, masseter hypertrophy, temporomandibular joint sounds—during routine consultations, to identify contributing factors such as psychological stress, sleep disturbance or medication use and to refer appropriately for dental or specialist assessment. A physician who has never heard the term is unlikely to elicit these features, so the deficit documented here translates into a real risk of under‐recognition of bruxism and of associated temporomandibular disorders in primary care, with delayed referral and avoidable progression of tooth wear or pain. Equipping medical graduates with basic competence in recognising bruxism and its associations would strengthen the primary‐care screening role that the 2025 consensus framework implicitly assigns to subject‐based and clinically based assessment [2], and provides the substantive justification for the curricular recommendation made below.

Self‐reported awareness of awake bruxism (36.1%) exceeded that of sleep bruxism (14.4%) in this cohort—a pattern that should be interpreted cautiously since awareness of one’s own waking behaviour is mechanistically easier to self‐detect than sleep‐time activity, which typically requires a bed partner or clinical examination to identify. The more conservative composite screening prevalence of 9.3% (95% CI: 6.5%–13.2%) sits toward the lower end of the 8%–31% range reported for adults in systematic review [6] and below figures reported among Saudi (42.3%), Italian (37.9% awake, 31.8% sleep) and Serbian (34.8%) student cohorts [8, 11, 12], but broadly comparable to the Nigerian undergraduate estimate of 24.7% once differing screening thresholds are accounted for [14]. No significant socio‐demographic differences in bruxism prevalence were observed in this study, consistent with mixed findings on sex differences reported in the wider literature [19].

The most frequently endorsed supportive sign was linea alba buccalis (19.6%), followed by TMJ clicking (12.4%)—both recognised, if non‐specific, clinical correlates of parafunctional activity reported at varying frequencies in other student populations [20, 21].

Perceived high stress was relatively uncommon in this cohort (8.9%), notably lower than the approximately one‐third prevalence reported among medical students globally [22] and among Saudi medical students specifically [23]. Despite this comparatively low overall stress burden, the significant positive correlation between stress and bruxism symptoms (Spearman’s *ρ* = 0.212; Pearson’s *r* = 0.25, 95% CI: 0.14–0.35) is consistent with the broader literature linking psychosocial stress to bruxism [8, 12, 24] and lends support to a model in which stress and bruxism may reinforce one another over time, although the cross‐sectional design precludes any claim of causal direction.

### 4.1. Strengths and Limitations

This study has several strengths. It provides the first peer‐reviewed Ghanaian data on bruxism knowledge and prevalence and their relationship with stress and, to our knowledge, only the second such dataset from West Africa. The sample size was determined a priori, and the stratified design with proportional allocation by course and clinical year—combined with random selection within strata—limits the selection bias that commonly affects convenience‐sampled student surveys. Stress was measured with a validated 30‐item instrument rather than a single global item, and bruxism status was derived from a multi‐item composite score rather than a single self‐report question, with all prevalence estimates now reported with CIs to permit formal comparison with other populations.

Several limitations must nonetheless be acknowledged. This study is limited by its reliance on self‐reported, subject‐based assessment of bruxism, which is susceptible to recall and reporting bias and, under the 2025 consensus framework, cannot be equated with clinically based or device‐based assessment; the absence of polysomnographic or electromyographic confirmation; the possibility of self‐selection and non‐response bias inherent in web‐based surveys, notwithstanding the response rate of (xx)%; the small dental‐student subgroup, which widens the CIs around dental‐specific estimates; the length of the 30‐item PSQ, which may have caused respondent fatigue; and the near‐total absence of prior published Ghanaian data against which to benchmark these findings. The cross‐sectional design also precludes inference about the direction of the stress–bruxism relationship.

## 5. Conclusion and Recommendations

Knowledge of bruxism among clinical‐year dental and medical students at the University of Ghana is generally poor, particularly among medical students and those in their earlier clinical years. Self‐reported bruxism symptoms are present in a meaningful minority of students, and although high perceived stress was uncommon in this cohort, stress was significantly and positively associated with bruxism symptom scores.

We recommend (1) explicit inclusion of bruxism aetiology, recognition and management in the medical curriculum, so that future physicians can fulfil a primary screening and referral role, alongside reinforcement within dental training; (2) routine screening of clinical‐year students for bruxism signs and symptoms, to enable early counselling or referral; (3) institutional stress‐reduction initiatives—such as structured wellness programming, peer support and manageable workload design—targeted at clinical‐year cohorts; and (4) further, ideally longitudinal, Ghanaian research using subject‐based, clinically based and device‐based assessment to clarify the direction of the stress–bruxism relationship.

## Funding

No external funding was received for this study.

## Ethics Statement

This study was approved by the Ethical Review Board, College of Health Sciences, University of Ghana. Informed consent was obtained from all participants.

## Conflicts of Interest

The authors declare no conflicts of interest.

## Supporting Information

Additional supporting information can be found online in the Supporting Information section.

## Supporting information


**Supporting Information** Supporting File S1: Study questionnaire (bruxism knowledge and symptom modules and the Perceived Stress Questionnaire).

## Data Availability

The dataset analysed during the current study is available from the corresponding author upon reasonable request.
