# Supplementary material for: Knowledge and Prevalence of Bruxism and Its Association With Stress Among Clinical‐Year Dental and Medical Students at the University of Ghana
Source: Int J Dent. 2026 Oct 15;2026:2469933. doi: 10.1155/ijod/2469933 (PMC13670201; doi:10.1155/ijod/2469933)
Supplement: Supplementary file 1 [file IJOD-2026-2469933-s001.docx]

**Supporting Information — File S1**

**Study Questionnaire**

Knowledge and Prevalence of Bruxism and Its Association With Stress Among Clinical-Year Dental and Medical Students at the University of Ghana

*This file reproduces the questionnaire supplied by the investigators for the published study. Question wording and response options are retained; formatting has been standardized for readability.*

Name (Initials)………………….

# Section A: Demography

**1. Age (years)…..**

**2. Sex**

Male	[ ]

Female [ ]

**3. Course of Study:**

Dentistry	[ ]

Medicine	[ ]

**4. Clinical Year:**

1st (Class of 2026)	[ ]

2nd (Class of 2025)	[ ]

Final year (class of 2024)	[ ]

# Section B: Knowledge of Bruxism

*Select the appropriate response(s) for each of the following questions*

**1. Have you heard of bruxism?**

Yes	[ ]

No	[ ]

*If yes, kindly go to questions 2 and 3 (select as many as apply). If no, kindly go to question 4*

**2. Bruxism is defined as…………….**

The clenching of one's jaws only	[ ]

The grinding of one's teeth either when awake or asleep	[ ]

The clenching of one's jaws and grinding of teeth either when awake or asleep. [ ]

If other, kindly specify …………………………………………………….

**3. Source of information about bruxism (select as many as apply)**

Mass media	[ ]

Peers	[ ]

Family	[ ]

School	[ ]

Health personnel [ ]

If other, kindly specify……….

**4. Do you know about the causes of bruxism?**

Yes	[ ]

No	[ ]

*If yes, kindly go to question 5 (select as many as apply). If no, kindly go to question 6.*

**5. If yes, what are they?**

Genetic factors	[ ]

Stress	[ ]

Malocclusion	[ ]

Side effects of certain drugs	[ ]

Psychiatric problems	[ ]

If other, kindly specify ………………………………………………………..

**6. Do you know of some of the consequences of bruxism?**

Yes	[ ]

No	[ ]

*If yes, kindly go to question 7 (select as many as apply). If no, kindly go to question 8*

**7. If yes, what are they?**

Tooth wear	[ ]

Myofascial pain	[ ]

Mobile teeth	[ ]

Tooth loss	[ ]

Insomnia	[ ]

Arthritis of the jaw	[ ]

If other, kindly specify……………………………………………….

**8. Do you know how this condition is treated?**

Yes	[ ]

No	[ ]

*If yes, kindly go to question 9 (select as many as apply). If no, kindly go to the next section.*

**9. If yes, how is it treated?**

With medication	[ ]

Adjustment of occlusion	[ ]

With dental guards and occlusal splints [ ]

If other, kindly specify…………………………………..

# Section C. Awareness and Experience of Bruxism

*Select the appropriate response for each of the following questions*

**1. Have you ever been aware of grinding or clenching your teeth during wakefulness before?**

Yes	[ ]

No	[ ]

**2. Are you aware or has anyone heard you grinding your teeth frequently during sleep?**

Yes	[ ]

No	[ ]

**3. Do you feel a sensation of fatigue, tightness, or soreness in your jaw upon waking from sleep?**

Yes	[ ]

No	[ ]

**4. Do you have pain in your facial muscles upon waking from sleep?**

Yes	[ ]

No	[ ]

**5. Do you feel your teeth are clenched or your mouth is sore upon waking from sleep?**

Yes	[ ]

No	[ ]

**6. Do you have a white line on the inside of your cheek?**

Yes	[ ]

No	[ ]

**7. Do you have aching in your temples upon waking from sleep?**

Yes	[ ]

No	[ ]

**8. Do you have difficulty opening your mouth wide upon waking from sleep?**

Yes	[ ]

No	[ ]

**9. Do you have a clicking sound in your jaw joint?**

Yes	[ ]

No	[ ]

**10. Do you feel tension in your joint upon awakening and feel as if you have to move your lower jaw?**

Yes	[ ]

No	[ ]

# Section D: Perceived Stress Questionnaire (PSQ)

For each sentence, select the number that describes how often it applies to you in general, during the last year or two.

For each statement, select one response: 1 = Rarely; 2 = Sometimes; 3 = Often; 4 = Usually.

| Statement | Rarely (1) | Sometimes (2) | Often (3) | Usually (4) |
| --- | --- | --- | --- | --- |
| 1. You feel rested | ☐ | ☐ | ☐ | ☐ |
| 2. You feel that too many demands are being made on you | ☐ | ☐ | ☐ | ☐ |
| 3. You are irritable or grouchy | ☐ | ☐ | ☐ | ☐ |
| 4. You have too many things to do | ☐ | ☐ | ☐ | ☐ |
| 5. You feel lonely or isolated | ☐ | ☐ | ☐ | ☐ |
| 6. You find yourself in situations of conflict | ☐ | ☐ | ☐ | ☐ |
| 7. You feel you're doing things you like | ☐ | ☐ | ☐ | ☐ |
| 8. You feel tired | ☐ | ☐ | ☐ | ☐ |
| 9. You fear you may not manage to attain your goals | ☐ | ☐ | ☐ | ☐ |
| 10. You feel calm | ☐ | ☐ | ☐ | ☐ |
| 11. You have too many decisions to make | ☐ | ☐ | ☐ | ☐ |
| 12. You feel frustrated | ☐ | ☐ | ☐ | ☐ |
| 13. You are full of energy | ☐ | ☐ | ☐ | ☐ |
| 14. You feel tense | ☐ | ☐ | ☐ | ☐ |
| 15. Your problems seem to be piling up | ☐ | ☐ | ☐ | ☐ |
| 16. You feel you're in a hurry | ☐ | ☐ | ☐ | ☐ |
| 17. You feel safe and protected | ☐ | ☐ | ☐ | ☐ |
| 18. You have many worries | ☐ | ☐ | ☐ | ☐ |
| 19. You are under pressure from other people | ☐ | ☐ | ☐ | ☐ |
| 20. You feel discouraged | ☐ | ☐ | ☐ | ☐ |
| 21. You enjoy yourself | ☐ | ☐ | ☐ | ☐ |
| 22. You are afraid for the future | ☐ | ☐ | ☐ | ☐ |
| 23. You feel you're doing things because you have to not because you want to | ☐ | ☐ | ☐ | ☐ |
| 24. You feel criticized or judged | ☐ | ☐ | ☐ | ☐ |
| 25. You are lighthearted | ☐ | ☐ | ☐ | ☐ |
| 26. You feel mentally exhausted | ☐ | ☐ | ☐ | ☐ |
| 27. You have trouble relaxing | ☐ | ☐ | ☐ | ☐ |
| 28. You feel loaded down with responsibility | ☐ | ☐ | ☐ | ☐ |
| 29. You have enough time for yourself | ☐ | ☐ | ☐ | ☐ |
| 30. You feel under pressure from deadlines | ☐ | ☐ | ☐ | ☐ |

Source cited in the original questionnaire: Levenstein et al. (1993).
